# Effects of Pituitary Surgery and High-Dose Cabergoline Therapy on Metabolic Profile in Patients With Prolactinoma Resistant to Conventional Cabergoline Treatment

**DOI:** 10.3389/fendo.2021.769744

**Published:** 2021-11-30

**Authors:** Rosa Pirchio, Renata S. Auriemma, Domenico Solari, Mauro Arnesi, Claudia Pivonello, Mariarosaria Negri, Cristina de Angelis, Luigi M. Cavallo, Paolo Cappabianca, Annamaria Colao, Rosario Pivonello

**Affiliations:** ^1^ Dipartimento di Medicina Clinica e Chirurgia, Sezione di Endocrinologia, Università “Federico II” di Napoli, Naples, Italy; ^2^ Department of Neuroscience, Reproductive Science and Odontostomatology, University of Naples Federico II, Naples, Italy; ^3^ Unesco Chair for Health Education and Sustainable Development, “Federico II” University, Naples, Italy

**Keywords:** prolactin, hyperprolactinemia, pituitary neurosurgery, cabergoline, high dose cabergoline, insulin resistance, lipid metabolism, pituitary tumors

## Abstract

**Objective:**

Control of prolactin excess is associated with the improvement in gluco-insulinemic and lipid profile. The current study aimed at investigating the effects of pituitary surgery and medical therapy with high dose cabergoline (≥2mg/week) on metabolic profile in patients with prolactinoma resistant to cabergoline conventional doses (<2mg/week).

**Design:**

Thirty-four patients (22 men, 12 women, aged 33.9 ± 12.5 years) with prolactinoma (4 microadenomas and 30 macroadenomas) were included in the present study. Among them 17 (50%) received pituitary surgery (PS, Group1) and 17 (50%) medical therapy with high dose cabergoline (Group 2).

**Methods:**

In the whole patient cohort, anthropometric (weight, BMI) and biochemical (fasting glucose and insulin, triglycerides, total, HDL and LDL-cholesterol, HOMA-IR, HOMA-β and ISI0) parameters were evaluated before and within 12 months after treatment.

**Results:**

In Group 1, prolactin (p=0.002), total cholesterol (p=0.012), and triglycerides (p=0.030) significantly decreased after pituitary surgery compared to the baseline. Prolactin significantly correlated with fasting glucose (r=0.056, p=0.025). In Group 2, fasting insulin (p=0.033), HOMA-β (p=0.011) and ISI0 (p=0.011) significantly improved compared to baseline. Postoperative cabergoline dose significantly correlated with Δfasting glucose (r=-0.556, p=0.039) and ΔLDL cholesterol (r=- 0.521, p=0.046), and was the best predictor of ΔLDL cholesterol (r^2 =^ 0.59, p=0.002) in Group 1.

**Conclusions:**

The rapid decrease in PRL levels induced by PS might improve lipid metabolism, whereas HD-CAB might exert a beneficial impact on both insulin secretion and peripheral sensitivity, thus inducing a global metabolic improvement.

## Introduction

Prolactin (PRL) exerts a wide variety of actions on metabolic profile in addition to the effects on gonadic function (1–4). Regardless from its etiology, hyperprolactinemia is known to influence the orexigenic-anorexigenic systems that regulate appetite (1–6), and to increase food intake and weight gain, thus leading to obesity (1–6), likely as a consequence of the functional blockade of dopaminergic tone (1–7). This provides the reason why metabolic disorders are often encountered in patients with chronic PRL excess. Particularly, hyperprolactinemia is associated with disorders of glucose and insulin metabolism (1–4, 8, 9), clinically translated in impaired glucose tolerance, insulin resistance, and postprandial hyperinsulinemia (1–4, 8, 9) together with reduced insulin sensitivity (10) either in obese or non-obese patients. *In vitro* studies on primary cultures of isolated rat pancreatic cells have documented that PRL excess results in enhanced β-cells replication (1–4, 8) and inappropriate increase in insulin production at fasting and after glucose load (1–4, 8). On the other hand, PRL may directly modulate adipose tissue function. In rats, PRL receptors increase during adipocyte differentiation, thus suggesting a potential direct influence on lipid metabolism in mature adipose cells (1–3, 11, 12).

Hyperprolactinemic patients often display an unfavorable lipid profile (1, 2) generally characterized by increased total and LDL cholesterol, triglycerides and apolipoprotein B, and decreased HDL cholesterol, apolipoprotein A-I and A-II as compared to healthy controls (1, 2). Notably, the expression of dopamine receptors type 2 (D2DR) on human pancreatic β-cells (13) and adipocytes (14) provided the rationale to investigate the effects of treatment with dopamine and dopamine agonists (DA), mainly bromocriptine (BRC) and cabergoline (CAB), known to represent the treatment of choice for patients with hyperprolactinemia (15, 16), on gluco-insulinemic and lipid metabolism. In diabetic patients, BRC, as quick release formulation, has been shown to exert a significant beneficial impact on fasting plasma glucose, glycated hemoglobin (HbA1c), and body weight reduction; on this basis, it has been officially approved as adjunctive glucose lowering therapy in patients with inadequately controlled type 2 diabetes mellitus (17). Similarly, CAB has been demonstrated to reduce fasting plasma glucose and HbA1c in diabetic subjects with suboptimal glycemic profiles being treated with different anti-diabetic drugs (18).

In patients with prolactinomas, treatment with CAB has been demonstrated to significantly reduce body weight, BMI, and waist circumference (19) and to ameliorate glucose profile and insulin resistance (19–21), CAB dose being directly correlated with such metabolic improvement rather than the correction of PRL excess (19, 20) or concomitant hypogonadism (22, 23). The restoration of normal prolactin values using CAB has also been demonstrated to be associated with significant improvement of adipose tissue disfunction evaluated as visceral adipose index (VAI) (19). Similarly, both BRC and CAB have been demonstrated to significantly improve lipid profile independently on their impact on concomitant obesity (19, 20, 22, 23) and hypogonadism (23), leading to the hypothesis of a direct beneficial effect of DA on lipid profile (19, 23). Noteworthy, all these studies have been performed in patients well responders to long-term treatment with DA at conventional doses, namely patients who achieved PRL normalization and a concomitant reduction of at least 50% in tumor volume (15, 16). Conversely, the metabolic characteristics of hyperprolactinemic patients resistant to DA have been scantly investigated. This might be explained considering that DA, mainly CAB, are generally effective in suppressing PRL levels and shrinking tumor mass in the vast majority of cases, with complete resistance occurring in 10% of patients with microprolactinoma and in less than 20% of those with macroprolactinoma treated with CAB (24).

Aside from DA, prolactinomas may benefit from pituitary surgery (PS), which is recommended in patients resistant to high dose treatment or intolerant to medical therapy or with severe optic chiasm compression and visual field defects (16). Whether surgical treatment of prolactinomas produces beneficial effects on metabolic alterations as DA is yet to be elucidated. Glucose and lipid metabolism have been investigated in patients with prolactinomas before and 4-6 months after PS (25). Noteworthy, the response of glucose and insulin during oral glucose tolerance test was significantly decreased compared to baseline (25); the reduction of insulin response was ascribed not only to the lower glucose levels but also to the increased insulin sensitivity as shown by the reduction of insulinogenic index (25). Conversely, fasting glucose, fasting insulin, total cholesterol and triglycerides remained unchanged (25).

To date, no study has investigated metabolic disorders in patients with prolactinomas resistant to conventional CAB dose treatment (i.e., <2 mg/week) (26), and no data are nowadays available about the metabolic effects of the alternative therapeutic approaches in such patients, like high dose CAB treatment (≥2 mg/week, HD-CAB) and PS. The current study aimed at investigating the effects of HD-CAB and PS on gluco-insulinemic and lipid metabolism in patients with prolactinomas resistant to conventional CAB treatment.

## Patients and Methods

### Inclusion and Exclusion Criteria

This prospective study included patients with an established diagnosis of prolactinoma resistant to CAB conventional dose, defined as patients not achieving complete PRL normalization at a CAB dose <2 mg per week, as previously reported (26). Inclusion criteria were: 1. age >18 years, and 2. diagnosis of prolactinoma resistant to CAB conventional dose. Exclusion criteria were represented by the presence at the study entry of the following conditions: 1. menopause; 2. hyperprolactinemia- induced hypogonadism; 3. hypopituitarism without or with replacement treatments; 4. PRL and GH co-secreting pituitary tumors; and 5. Type 2 diabetes mellitus and/or dyslipidemia receiving medical treatment. Patients with incomplete data and those who became pregnant while on treatment were not considered for the final analysis of the study. The patients included in the study provided a written informed consent with respect to a confidentiality statement of data collection according to the Italian privacy policy.

### Patients

Forty-one consecutive adult patients with prolactinoma resistant to CAB conventional dose attended the outpatient clinic of Neuroendocrine Disease Unit at ‘Federico II’ University of Naples between January 2017 and December 2018. Pituitary imaging revealed a microadenoma in 6 patients and a macroadenoma in 35 patients. Five patients did not enter the study because of menopause in 2 women (4,8%), and hypopituitarism in 3 men (7,3%), requiring replacement treatment with corticosteroids and testosterone in 3 and levothyroxine in 2 patients, respectively. Two patients were excluded from the analysis because of gestation occurring while on therapy (4,8%). Therefore, 34 patients (22 men,12 women, aged 33.9 ± 12.5 years), the totality bearing a pituitary adenoma (4 with microadenoma and 30 with macroadenoma, including 6 with giant tumors, defined as a tumor diameter greater of 4 cm in size), were considered for the study. In the whole patient cohort, IGFI levels were evaluated before and after treatment with PS or HD-CAB in order to identify and exclude from the current analysis all patients developing subclinical or overt growth hormone deficiency; however, no patient showed a decrease in IGF-I levels and/or developed growth hormone deficiency throughout the study. Among women, none received estrogen replacement and/or oral contraceptives throughout the study. Patient profile at study entry is shown in **Table 1**. In the whole patient cohort none has received radiotherapy before study entry and throughout the study.

**Table 1:** Patient profiles at study entry.

*Number*	34
***Age, years***	33.9±12.5 years
***Male/female***	22/12
***Microadenoma, n (%)***	4 (11,7)
***Macroadenoma, n (%)***	30 (88,3)
***Giant tumors, n (% of Macroadenoma)***	6 (20)
***PRL level, μg/l***	719.7±2343.61

### Study Protocol

The present is a prospective study. At diagnosis and thereafter at 3- to 6-month intervals, all patients were admitted to the hospital for a complete biochemical and endocrine examination. At each time point biochemical parameters, including fasting glucose (FG) and fasting insulin (FI), serum triglycerides (TG), total (TC), HDL and LDL cholesterol were evaluated. On the basis of the plasma glucose levels the diagnosis of impaired glucose tolerance and diabetes mellitus was performed according to WHO guidelines (27). Insulin resistance was assessed using the HOMA index in line with Matthews et al. (28), by calculating the HOMA-IR= [insulin (mU/l) × FG (mmol/l)]/22.5 as surrogate index of insulin resistance, and the HOMA-β= [20 × insulin (mU/l)/FG (mmol/l) –3.5] as surrogate index of insulin secretion (28). In order to assess baseline insulin sensitivity, the ISI0 was calculated according to the following formula (29): ISI0 = 10,000/insulin (μU/ml) × FG (mg/dl). Serum PRL levels were assessed in all patients at diagnosis and every 3–6 months during the following period. Blood samples were collected between 07: 00–08: 00 h after an overnight fasting. This study considered two time points: baseline and post-treatment evaluation (within 12 months post treatment).

### Treatment Protocol

According to the standard protocol of the center (30, 31), in patients with microprolactinomas CAB was administered orally at a starting dose of 0.25 mg twice weekly for the first 2 weeks and then 0.5 mg twice weekly. Dose adjustment was carried out every 3–6 months on the basis of serum PRL levels. In patients with macroprolactinomas, CAB was administered at a starting dose of 0.25 mg once a week for the first week and then twice weekly. Dose adjustment was performed at 3- to 6- month intervals on the basis of serum PRL levels. In patients who did not normalize PRL levels, CAB dose was progressively increased up to 2 mg/week, and in those who did not normalize even with 2 mg/week, PS was proposed. In patients spontaneously refusing PS, HD-CAB was used in order to overcome resistance to CAB conventional treatment. Based on final treatment, patients were classified as Group 1, including patients who underwent trans-sphenoidal PS (17 patients, 50%), and Group 2, including patients receiving HD-CAB (17 patients, 50%).

### Assays

Glucose and lipid levels were measured by standard methods. Insulin and PRL levels were measured by chemiluminescent immunometric assay using commercially available kits (Immulite DPC, Llamberis, UK). For insulin, the sensitivity was 4 mU/ml, the intra-assay coefficients of variation (CV) were 5.5–10.6%, and the corresponding inter-assay CV values were 6.2–10.8%. For PRL, the sensitivity was 0.16 μg/l, the intra-assay CV values for PRL concentrations of 22 and 164 μg/l were 2.3 and 3.8%, respectively, and the corresponding inter-assay CV values were 6%. Normal PRL levels were 5–25 μg/l in women and 5–20 μg/l in men. Hyperprolactinemia was defined as a serum PRL level >25 μg/l on two different samples taken after an interval longer than 1 week.

### Statistical Analysis

Data were analyzed using SPSS Software for Windows, version 24 (SPSS, Inc., Cary, N.C., USA). Data are reported as mean ± SD, unless otherwise specified. The comparison between the numerical data before and after treatment with PS or HD-CAB was made by non-parametric Wilcoxon test for continuous variables. The comparison between the numerical data between the two different groups of patients was made by non-parametric U Mann-Whitney test. The correlation study was done by calculating Pearson’s correlation coefficients. Regression analysis was performed to investigate the best predictors of metabolic improvement in the present patient cohort. Significance was set at 5%.

## Results

The metabolic and hormonal parameters in the whole patient cohort at baseline and within 12 months of treatment are shown in **Table 2**.

**Table 2:** Effects of treatment with surgery and HD-CAB (Group 1) vs. HD-CAB alone (Group 2) on disease control and metabolic parameters.

	Group 1			Group 2				
***M/F***	10/7	–	–	12/5	–	–	–	–
***Micro/macro***	2/15	–	–	2/15	–	–	–	–
	**Baseline (A)**	**12 months (B)**	**p (A vs. B)**	**Baseline (C)**	**12 months (D)**	**p (C vs. D)**	**p (A vs. C)**	**p (B vs. D)**
***PRL level, μg/l***	**1354.84±3396.05**	**77.24±107.84**	**0.002**	249.02±422.03	166.43±255.37	0.136	0.779	0.214
***Fasting glucose, mg/dl***	87.48±13.01	89.06±10.33	0.576	94.64±16.7	92.3±15	0.492	0.289	0.732
***Fasting insulin, mU/l***	16.19±9.0	12.97±7.57	0.330	**20.81±2.59**	**16.41±19.14**	**0.033**	0.674	0.428
***Total cholesterol, mg/dl***	**204.00±39.51**	**179.43±29.2**	**0.012**	198.23±29.5	190.7±36.6	0.196	0.928	0.256
***HDL cholesterol, mg/dl***	49.37±10.07	47.5±8.4	0.660	50.53±15.81	48.66±15.3	0.368	0.968	0.759
***Triglycerides, mg/dl***	**153,75±98,04**	**106.7±56.62**	**0.030**	137.41±61.3	135.88±110.04	0.352	0.589	0.402
***LDL cholesterol, mg/dl***	124.14±28.4	112.36±25.57	0.196	123.6±24.43	115.33±38.01	0.301	0.412	0.601
***HOMA-IR***	3.37±2.01	3.16±1.68	0.330	4.84±5.8	3.8±4.63	0.055	0.703	0.779
***HOMA-β***	297.41±262.50	208.42±95.70	0.233	**338.88±374.10**	**224.06±220.34**	**0.011**	0.610	0.779
***ISI0***	11.66±9.55	11.28±9.4	0.233	**8.97±5.8**	**11.53±7.34**	**0.011**	0.639	0.564
***Impaired Fasting Glucose, n (%)***	4 (23.5%)	2 (11.7%)	0.383	6 (35.3%)	5 (29.4%)	0.723	0.706	0.396
***Diabetes Mellitus, n (%)***	0 (0%)	0 (0%)	/	1 (5.88%)	1 (5.88%)	/	/	/

The bold values means statistically significant.

### Baseline Evaluation

At baseline, mean PRL levels were similar in both groups (p=0.413). Treatment duration with CAB before study entry was 13±22.1 months in Group 1 and 70.3±55.7 months in Group 2, respectively. Impaired fasting glucose (IFG) was found in 4 patients (23.5%) in Group 1 and in 6 patients (35.3%) in Group 2, whereas no patient in Group 1 and 1 patient (5.6%) in Group 2 had a new diagnosed diabetes mellitus (DM) not receiving glucose lowering treatment yet. At study entry no significant difference in anthropometric (weight, BMI) and metabolic (FG, FI, total, LDL and HDL cholesterol, TG, HOMA-IR, HOMA- β and ISI0) parameters between the two groups was found. HOMA-IR was >2.5 in 9 patients (53%) in Group 1 and in 11 patients (64.7%) in Group 2.

### Post-Treatment Evaluation

At post-treatment evaluation, no patient in Group 1 developed pituitary hormone deficiency following PS. In Group 2 CAB dose ranged 2-7 mg/week (median 3 mg/week). No significant difference was found in weight and BMI between the two groups. PRL levels were significantly reduced in Group 1 (p=0.002) and slightly reduced in Group 2 (p=0.136) compared to baseline. PRL fully normalized in 64.7% in Group 1 and 52.9% in Group 2, with no significant difference between the two groups (p=0.727). IFG was still confirmed in 2 patients (11.7%) in Group 1 (p=0.383), and in 5 patients (29.4%) in Group 2 (p=0.723), whereas the prevalence of DM did not change in both groups throughout the study. HOMA-IR was >2.5 in 11 patients (64.7%, p=0.730) in Group 1 and in 8 patients (47%, p=0.488) in Group 2. Regarding gluco-insulin parameters, in Group 1, no significant changes was found in FG, FI, HOMA-IR, HOMA-β and ISI0. Conversely, in Group 2 FI (p=0.033), HOMA-β (p=0.011) and ISI0 (p=0.011) significantly improved compared to baseline (**Table 2**). In this Group FG (p=0.492) was only slightly but not significantly reduced (**Table 2**).

Regarding lipid parameters, in Group 1 a significant decrease in TC (p=0.012) and TG (p=0.03) was found, whereas no significant difference was recorded in LDL and HDL cholesterol (**Table 2**). Conversely, in Group 2 a slightly but not significant change in lipid fractions was found compared to baseline (**Table 2**).

PS resulted in % decrease (Δ) in FG of 2.85%, FI of 8.84%, TC of 7.95%, TG of 8.8%, LDL of 8.8%, and in HDL increase of 1.1%. On the other hand, HD-CAB determined % decrease in FG of 0.96%, FI of 15%, in TC of 3.5%, in TG 1.43%, in LDL of 5.7% and resulted in % increase of 1.65% in HDL.

### Correlation Study

**Figure 1:**
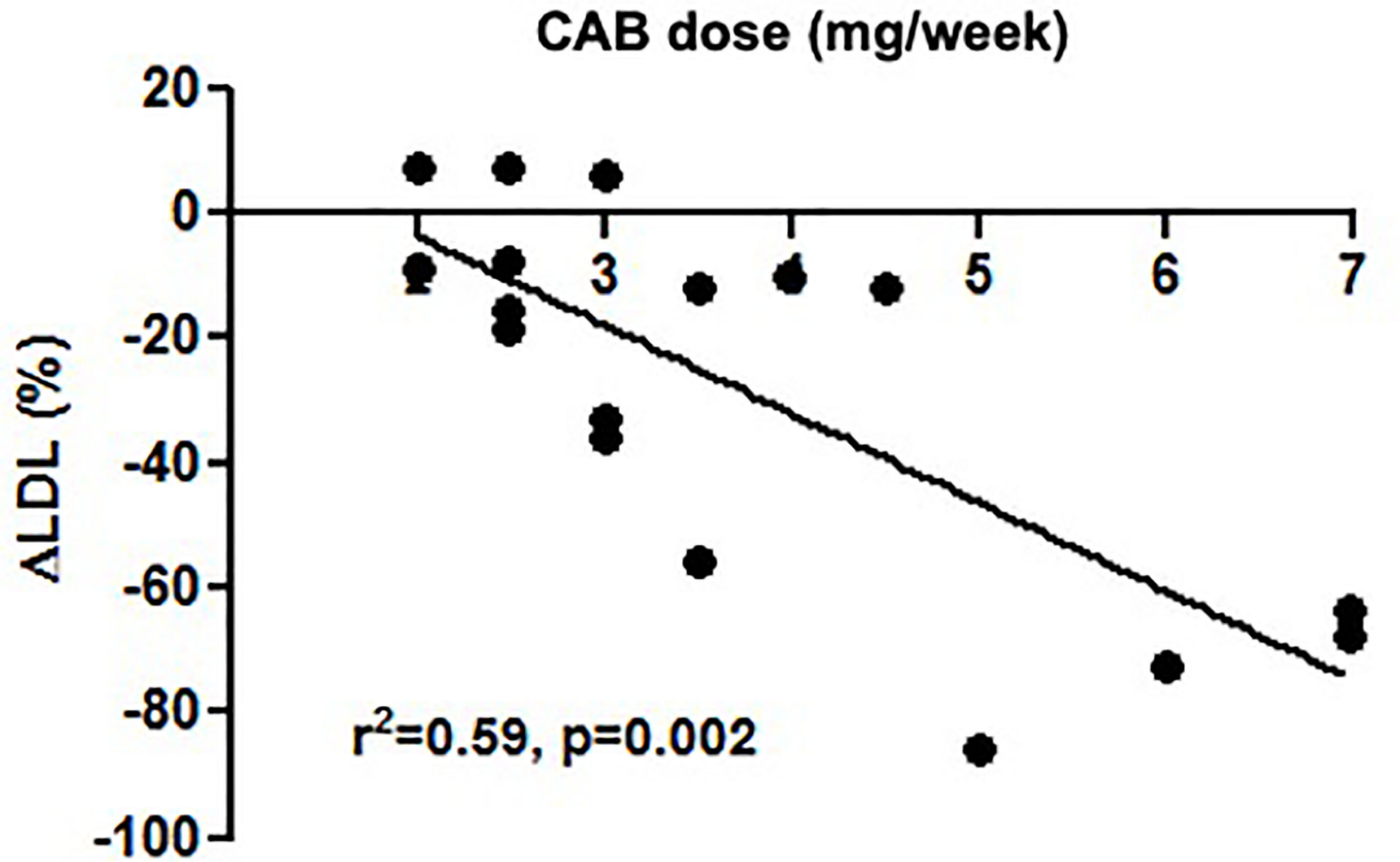
CAB dose was the best predictor of percent change (Δ%LDL) in Group 1.

At post-treatment evaluation, neither PRL nor CAB dose correlated with changes in weight and BMI. CAB dose significantly correlated with ΔFG (r=0.556, p=0.039) and ΔLDL cholesterol (r=0.521, p=0.046) in Group 1, and post-treatment PRL (r=0.709, p=0.001) in Group 2 (**Table 3** *)*. CAB dose was the best predictor of ΔLDL (r^2^ = 0.59, p=0.002) in Group 1 (**Figure 1**). Post-treatment PRL significant correlated with FG (r=0.556, p=0.025) in Group 1, and with TC (r=0.556, p=0.021) and LDL cholesterol (r=0.616, p=0.009) in Group 2 (**Table 3** *)*. PRL percent change (Δ%) significantly correlated with ΔFG (r=0.674, p=0.004) in Group 1, and similarly with ΔFG (r=-0.590, p=0.013) and ΔHDL (r=-0.499, p=0.042) in Group 2 (**Table 3** *)*. Treatment duration with CAB before study entry was not correlated to Δ% in FG, FI, TC, TG, HDL and LDL either in Group 1 or in Group 2.

**Table 3:** Correlation study: impact of cabergoline dose and prolactin levels on metabolic parameters and their % change through the study.

	Group 1	r	p	Group 2	r	p
***Cabergoline dose***	Prolactin	0.246	0.358	Prolactin	**0.709**	**0.001**
	Fasting glucose	0.276	0.302	Fasting glucose	-0.144	0.582
	Fasting insulin	0.169	0.438	Fasting insulin	-0.161	0.537
	Total cholesterol	-0.279	0.296	Total cholesterol	0.283	0.272
	HDL cholesterol	-0.240	0.370	HDL cholesterol	-0.173	0.508
	LDL cholesterol	-0.169	0.531	LDL cholesterol	0.361	0.155
	Triglycerides	-0.019	0.945	Triglycerides	-0.042	0.872
***Prolactin***	Fasting glucose	**0.556**	**0.025**	Fasting glucose	-0.120	0.647
	Fasting insulin	0.275	0.387	Fasting insulin	-0.227	0.381
	Total cholesterol	-0.092	0.734	Total cholesterol	**0.556**	**0.021**
	HDL cholesterol	0.471	0.065	HDL cholesterol	-0.035	0.895
	LDL cholesterol	-0.308	0.245	LDL cholesterol	**0.616**	**0.009**
	Triglycerides	0.309	0.245	Triglycerides	-0.075	0.775
***Cabergoline dose***	% change prolactin	-0.354	0.215	% change prolactin	-0.148	0.571
	% change fasting glucose	**-0.556**	**0.039**	% change fasting glucose	0.004	0.987
	% change fasting insulin	0.001	0.998	% change fasting insulin	0.121	0.645
	% change total cholesterol	-0.344	0.228	% change total cholesterol	-0.385	0.127
	% change HDL cholesterol	-0.189	0.518	% change HDL cholesterol	0.086	0.742
	% change LDL cholesterol	**-0.521**	**0.046**	% change LDL cholesterol	-0.300	0.242
	% change Triglycerides	-0.258	0.373	% change Triglycerides	0.194	0.455
***% change prolactin***	% change fasting glucose	**0.674**	**0.004**	% change fasting glucose	**-0.590**	**0.013**
	% change fasting insulin	0.183	0.497	% change fasting insulin	-0.142	0.586
	% change total cholesterol	0.168	0.533	% change total cholesterol	-0.369	0.145
	% change HDL cholesterol	-0.308	0.245	% change HDL cholesterol	**-0.499**	**0.042**
	% change LDL cholesterol	-0.459	0.074	% change LDL cholesterol	-0.070	0.790
	% change Triglycerides	0.396	0.129	% change Triglycerides	-0.144	0.582

The bold values means statistically significant.

## Discussion

The results of the current study firstly demonstrated that in patients with prolactinoma resistant to CAB conventional dosing both PS and HD-CAB significantly impact disease control and improve gluco-insulinemic and lipid profile, although with different results.

Whether metabolic improvement seen after treatment in patients with hyperprolactinemia reflects the beneficial effects of PRL lowering or of CAB administration is still debated. PRL excess and functional blockade of dopaminergic tone are key mechanisms implied in the pathogenesis of weight gain and obesity frequently described in patients with prolactinomas (5–7). Previous investigations have shown the decrease in body weight, BMI, and body fat percentage to occur in patients with prolactinomas after the achievement of PRL normalization following DA treatment (5, 32), and a direct relationship between such an improvement and D2DR activation has been proposed (22). In the current study, no significant change was found in weight and BMI in the two groups of patients, thus leading to the conclusion that neither the rapid reduction in PRL levels induced by PS nor the prolonged exposure to HD-CAB treatment schedule exerted a significant impact on anthropometric parameters in prolactinomas. Previous evidence has demonstrated that the sustained and prolonged PRL normalization following CAB treatment for 5 years resulted in the significant reduction of body weight and BMI (19). As a consequence, a significant improvement in gluco-insulinemic and lipid profile has been reported in those patients fully responsive to CAB therapy (19). Current results might be explained, at least partly, considering that prior to the study entry patients had received long-term CAB therapy but with modest biochemical efficacy, and PRL normalization occurred approximately in two thirds of patients receiving PS and in half of those medically treated with HD- CAB. Furthermore, short-term CAB treatment (up to 12 months) has been shown not to induce a significant decrease in body weight and BMI (19), as in the current study, thus suggesting that the impact on body weight and BMI might require a longer treatment or follow-up. Consistently with these findings, previous investigations have demonstrated a scant effect of 6-month therapy with CAB on weight and BMI (33)

The existence of a correlation between PRL excess and hyperinsulinemia, glucose abnormalities and DM, is a matter of fact, and correction of hyperprolactinemia has been shown to ameliorate gluco-insulinemic dysfunction (1, 19, 20, 23). *In vitro* insulin secretion has been demonstrated to be enhanced by PRL (1–4, 8) and suppressed by D2DR activation (1, 8, 13). In patients with chronic hyperprolactinemia treated with CAB for at least 6 months the significant reduction in FG and HOMA- IR has been demonstrated (33). Longer CAB treatment up to 5 years has resulted in the significant decrease in FI and HOMA-IR regardless of changes in body weight and BMI (19). The findings of the current study have shown that FI, HOMA-β and ISI0 improved in patients receiving HD-CAB up to 12 months, whereas gluco-insulinemic parameters only slightly improved in those treated by PS. Nevertheless, in both groups of treatment, percent change in PRL levels significantly correlated with changes in FG, thus suggesting a potential role for PRL reduction as glucose-lowering mechanism. However, consistently with previous results (19) the current study has confirmed CAB dose to significantly correlate with the improvement in glucose profile. Present results reinforce the hypothesis of a direct beneficial effect of CAB therapy on gluco-insulinemic levels, as well as on insulin secretion and peripheral sensitivity, rather than of PRL decrease *per se* (19, 23). In this light, the correlation between CAB dose and percent change in FG found in the current study support the potential application of CAB as glucose lowering drug, independently on PRL levels. On the other hand, these findings also confirm a previous experience reporting a modest and not significant reduction in FG, TC and TG in patients with prolactinomas undergone PS, that induced biochemical control in 58% of cases (25).

In line with this latter study (25), in the current investigation lipid profile significantly improved after PS, as TC and TG significantly decreased as compared to baseline although HDL and LDL cholesterol did not significantly ameliorate. Interestingly, in the present study HD-CAB did not significantly improve lipid fractions. These findings might be explained by considering the fine and complex interplay between the effects of treatments on PRL reduction at one side, and body weight on the other side. A direct correlation between PRL levels and lipid profile has been proposed (21), since adipocytes reportedly release PRL and express PRL receptors, known to influence differentiation of mature adipocytes (7). Increased TC, LDL and TG and reduced HDL cholesterol have been reported in patients with prolactinomas as compared to healthy control subjects (34–37). Noteworthy, adipocytes also express D2DR receptors, whose activation results in the inhibition of PRL expression and release (36). Whether therapy with CAB might beneficially impact lipid profile in patients with prolactinomas is still under investigation, and controversial results have been reported. In some studies (19, 20, 22, 38–40) long-term CAB has been shown to significantly improve lipid fractions and visceral adiposity index, i.e. adipose tissue dysfunction (19, 20), independently on concomitant changes in PRL levels and body weight, thus leading to the conclusion that CAB *per se* might improve lipid metabolism. Other studies have failed to demonstrate a similar effectiveness of CAB on lipid profile (32, 40), thus raising the question of whether such an improvement also requires concomitant PRL normalization and BMI decrease, aside from CAB direct effects (41). In line with this latter hypothesis, in the current study a significant improvement has been found in TC and TG following PS, with HD-CAB inducing only a slight reduction in lipid fractions. In patients surgically treated PRL significantly correlated with total and LDL cholesterol, whereas in those receiving HD- CAB percent decrease in PRL levels significantly correlated with changes in HDL, thus confirming that a rapid decrease in PRL levels might be necessary to improve lipid fractions. In turn, in patients medically treated CAB dose significantly correlated with percent decrease in LDL cholesterol, strengthening the hypothesis of a direct beneficial effect of CAB on adipose tissue function even independently on PRL normalization and changes in body weight.

In conclusion, these findings provide evidence that PRL levels and CAB dose strongly and mutually influence metabolic profile in patients with prolactinomas. The beneficial impact on gluco-insulinemic and lipid profile might reflect both PRL decrease and direct CAB effects. Even in absence of complete PRL normalization, the rapid decrease in PRL levels induced by surgical treatment might trigger a mechanism of lipid lowering, which apparently is not necessarily linked to weight loss. In turn, HD- CAB might display an intrinsic beneficial effect on gluco-insulinemic profile, mainly insulin secretion and peripheral sensitivity, independently on body weight and BMI amelioration. Future research will clarify the role and the burden of PRL levels and CAB dose on the improvement of metabolic profile in patients with prolactinomas resistant to CAB conventional dosing.

## Data Availability Statement

The raw data supporting the conclusions of this article will be made available by the authors, without undue reservation.

## Ethics Statement

Ethical review and approval was not required for the study on human participants in accordance with the local legislation and institutional requirements. The patients/participants provided their written informed consent to participate in this study.

## Author Contributions

RPir and RA conceived the study and prepared the manuscript drafting. RPir, RA, and DS performed literature search and contributed to the interpretation of the data. CP, MA, MN, and CA contributed to literature search prepared the tables and figure. RPir and RA wrote the manuscript. LC, PC, and AC provided a significant expert contribution in the scientific content revision process and revised it for important intellectual content. RPiv critically reviewed the manuscript. All authors contributed to the article and approved the submitted version.

## Funding

The study was funded by POR FESR CAMPANIA 2014 – 2020 “Diagnostic and therapeutic innovations for rare neuroendocrine, endocrine tumours and glioblastoma by an integrated technological platform of clinical, molecular, genomic, ICT, pharmacological and pharmaceutical expertise” RARE.PLAT.NET (to AC). The funder was not involved in the study design, collection, analysis, interpretation of data, the writing of this article or the decision to submit for publications.

## Conflict of Interest

CP received research grants from Corcept Therapeutics. AC has been Principal Investigator of Research Studies for Novartis, Ipsen, Pfizer, Lilly, Merck and Novo Nordisk; consultant for Novartis, Ipsen, Pfizer, and received honoraria from Novartis, Ipsen and Pfizer beyond the confines of this work. RPiv has been Principal Investigator of Clinical and/or Translational Research Studies for Novartis, HRA Pharma, Ipsen, Shire, Corcept Therapeutics, Cortendo AB, Janssen Cilag, Camurus, Strongbridge, and Pfizer; Co-investigator of Research Studies for Pfizer; received research grants from Novartis, Pfizer, Ipsen, HRA Pharma, Shire, IBSA, Strongbridge Biopharma; has been an occasional consultant for Novartis, Ipsen, Pfizer, Shire, HRA Pharma, Cortendo AB, Ferring, Strongbridge Biopharma, Recordati, Corcept Therapeutics, Crinetics Pharmaceuticals, ARH Healthcare, Biohealth Italia, Damor Farmaceutici, Italfarmaco; and has received fees and honoraria for presentations from Novartis, Shire, Pfizer and Recordati beyond the confines of this work.

The remaining authors declare that the research was conducted in the absence of any commercial or financial relationships that could be construed as a potential conflict of interest.

## Publisher’s Note

All claims expressed in this article are solely those of the authors and do not necessarily represent those of their affiliated organizations, or those of the publisher, the editors and the reviewers. Any product that may be evaluated in this article, or claim that may be made by its manufacturer, is not guaranteed or endorsed by the publisher.
